# Lymphocytic Esophagitis: A Rare Disease on the Rise

**DOI:** 10.7759/cureus.2153

**Published:** 2018-02-04

**Authors:** Lindsey C Shipley, Laith A Al Momani, Allison Locke, Mark Young

**Affiliations:** 1 Internal Medicine, Quillen College of Medicine, East Tennessee State University; 2 Internal Medicine, East Tennessee State University; 3 ETSU Gastroenterology, East Tennessee State University; 4 Gastroenterology Associates of Northeast Tennessee, East Tennessee State University

**Keywords:** esophagitis, lymphocytic esophagitis, perforation

## Abstract

Lymphocytic esophagitis is a rare, poorly understood disease. This case report presents a patient with a history of squamous cell carcinoma of the tongue who presented with dysphagia. He received esophageal dilation that unfortunately resulted in perforation. Biopsies showed lymphocytic esophagitis. There are very few cases in the literature describing perforation in lymphocytic esophagitis. In addition, management and treatment have been challenging for physicians; however, this case represents a complete symptomatic improvement in four to six weeks with a proton pump inhibitor.

## Introduction

Lymphocytic esophagitis (LE) is a rare disorder of the esophagus with most cases presenting with normal-appearing esophageal mucosa [1-2]. This case report discusses a patient with a history of post-radiation squamous cell carcinoma of the tongue, who presented with dysphagia due to recurrent esophageal strictures. Dilation, unfortunately, resulted in perforation.

## Case presentation

A 71-year-old male patient, with a known history of squamous cell carcinoma of the tongue treated with external beam radiation 17 years prior, presented to the clinic for esophagogastroduodenoscopy (EGD) with dilation for a follow-up for esophageal strictures. His only complaint at this time was dysphagia.

On prior EGD, he was noted to have a stricture in the upper esophagus, a ringed lower esophagus, and a lower esophageal stricture. His past medical history was significant for squamous cell carcinoma of the tongue status post radiation, gastroesophageal reflux disorder, and hypertension. Surgical history consisted of multiple EGDs requiring dilation with a French Maloney. A physical examination revealed no abnormal findings and laboratory testing was unremarkable.

**Figure 1:**
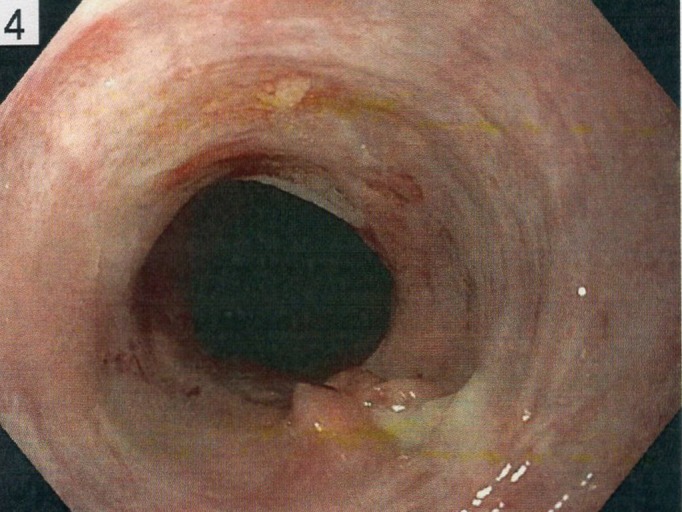
White streaking and stricture of esophagus via esophagogastroduodenoscopy

The patient underwent the procedure with dilation, at which time a characteristic white streaking and esophageal stricture were noted, as seen in Figure 1. However, this procedure was unfortunately complicated by an esophageal tear requiring six endoclips to close. The patient was admitted for observation, treated conservatively, and discharged the following day after a negative gastrografin esophagram. Biopsies were taken at the time of the procedure, which showed lymphocytic esophagitis with greater than 45 lymphocytes per high power field. This patient was treated with four-week proton pump inhibitor (PPI) therapy that resulted in complete symptom resolution.

## Discussion

Current treatment recommendations for lymphocytic esophagitis include symptomatic management similar to that in eosinophilic esophagitis or gastroesophageal reflux disease (GERD). However, there are no guidelines for the safety of endoscopic procedures in these cases. Combining both biopsy and dilation can increase the risk for perforation. We suggest delaying endoscopic dilation in such patients until symptoms have either resolved or improved.

Lymphocytic esophagitis (LE) is a rare disorder of the esophagus that was first reported in 2006 [3]. In a demographic study by Genta et al., only 116 patients were identified out of 129,252 [4]. The rise in the diagnosis of lymphocytic esophagitis could be due to the increase in endoscopic procedures and the availability of dedicated pathologists. LE is characterized by an increased number of intraepithelial lymphocytes without granulocytosis in the esophagus [5]. It appears to have a benign course in most patients and is most commonly found in older females and in younger patients with inflammatory bowel disease, specifically Crohn’s Disease. The most frequent endoscopic findings include esophageal rings, esophagitis, and esophageal strictures. Perforation is an uncommon complication of lymphocytic esophagitis and has only been documented in two other cases in the literature [2].

Intraepithelial lymphocytes in the esophagus are relatively common and the differential is broad, to include gastroesophageal reflux, lymphocytic esophagitis, changes associated with radiation therapy, and mucosal changes in motility disorders [2]. Diagnosis includes a biopsy with a high number of intraepithelial lymphocytes in the peripapillary areas (more than 35 lymphocytes per high power field) in addition to the absence of both eosinophils and granulocytes and severe spongiosis [5].

The management and treatment of symptomatic lymphocytic esophagitis are challenging for physicians, as there are no formal guidelines. Current treatment recommendations include symptomatic management similar to that in eosinophilic esophagitis or GERD, which includes proton pump inhibitors, swallowed topical steroids, and endoscopic dilation [3]. In addition, special precautions should be taken when performing endoscopic dilation, as a few cases have resulted in perforation [2].

## Conclusions

This case highlights a rare complication of lymphocytic esophagitis. We aim to make providers aware that these patients may be at greater risk for esophageal perforation. If lymphocytic esophagitis is suspected by endoscopy, and both biopsy and dilation are required, it may be in the patient's best interest to wait for dilation until they have received four to six weeks of proton pump inhibitors, to decrease the risk of perforation. However, more research is needed to determine the best approach for the management of patients with LE.
